# Letter to the editor regarding the article ‘individual and household risk factors of severe acute malnutrition among underfive children in Mao, Chad: a matched casecontrol study’

**DOI:** 10.1186/s13690-019-0336-2

**Published:** 2019-03-28

**Authors:** Sunanda Gupta, Kiran Goswami

**Affiliations:** Center for Community Medicine, All India Institute of Medical Sciences, New Delhi, 110029 India

**Keywords:** Malnutrition, Case-control studies, Severe acute malnutrition

## Abstract

We would like to thank the authors Jovana Dodos et al., for the article “Individual and household risk factors of severe acute malnutrition among underfive children in Mao, Chad: a matched casecontrol study”.

We would like to thank the authors Jovana Dodos et al., for the article “Individual and household risk factors of severe acute malnutrition among underfive children in Mao, Chad: a matched casecontrol study” [1], for addressing the age-old problem of childhood malnutrition that continues to embarrass us even today, despite magnificent progress in health sciences [2]. It aimed at identifying of household and individual risk factors in severe acute malnourished children of age 6–59 months in Mao, Chad. However few observations are being mentioned below to get clarity in solution to the problem.

The title suggests all under-five children but children of age between 6 to 59 months have been included as study population, as the problem is identified in this group.

The cases were selected from hospital with well-defined inclusion criteria and their anthropometric details were recorded from hospital register. It is assumed that anthropometric details in the hospital were taken by trained personnel, following standard procedures. Details of anthropometric measurement in the field have been well-described. We feel that study could be strengthened by following similar procedure at two places or at least providing details on quality of measurements done at hospital.

The vaccination criterion used was too stringent, with physical presence of vaccination card. This resulted in inability to study the variable question. We feel less stringent criterion like Bacille Calmette Guerine vaccine (BCG) scar or elicited history could have been used.

Despite meticulous method followed in designing the questionnaire, standard procedure of back-translation was not mentioned in the article.

Mid-upper arm circumference (MUAC) is a criterion for measuring nutritional status in children aged between 6 months and 5 years, but loses its value in adults. We feel that a more robust indicator such as body mass index (BMI) of caregiver could have been used.

We tried to recalculate sample size based on the assumptions clearly mentioned by author and realized that 10% loss due to non-response was taken but never mentioned in the article.

We are not clear from the article if more than one child from the same family, had been taken as cases or controls. If more than one child from same family was taken, a multi-level logistic regression analysis (hierarchial model) would have been more apt.

The individual risk factors can be confounders to household risk factors and vice-versa. We feel that a single model for statistical analysis would have sufficed to study these factors.

It is disheartening to plan and collect data for so many variables and then not being able to use them. For a stepwise selection procedure it would have been better to use a higher *p*-value threshold, such as 0.2 rather than 0.05 [3].

Sample size had been calculated considering Odds ratio to be 3.00. But in the results section, variables ‘not washing hands after defecation/using toilet’ and ‘absence of toilet in the household’, showed Odds ratio to be 1.9 reported as significant. Reporting of *p*-value of bivariate analysis would have given a better overview. Also, there are wide confidence intervals for adjusted odds ratio for several variables, due to low number of cases.

Though authors say that recall bias and social desirability bias are not possible because of their presence in both the groups, we feel that recall bias is possible in interviews of caregiver as detailed history was possibly taken taking for case management in the health center, just a day before. The exposure to health professionals is expected to alter their knowledge, thus leading to socially desirable answers.

In most of the communities, food available in family kitchen is recommended to be used as weaning food with due modification in consistency and spices. Hence there is nothing wrong in giving porridge as complementary food. No addition of oil/ sugar/ fats may be cause of it being less energy dense. We feel the variable selected should have been more specific with focus of feeding pattern, feeding habits (self/ by caregiver), distribution, consistency, and addition of oil/ sugar and fats etc. for any meaningful outcome for specific intervention. On the whole, the study promised much more but failed to achieve a lot.

## Abbreviations

BCG: Bacille Calmette Guerine vaccine
BMI: Body mass index
MUAC: Mid-upper arm circumference
